# Alternative Pathway Is Essential for Glomerular Complement Activation and Proteinuria in a Mouse Model of Membranous Nephropathy

**DOI:** 10.3389/fimmu.2018.01433

**Published:** 2018-06-22

**Authors:** Wentian Luo, Florina Olaru, Jeffrey H. Miner, Laurence H. Beck, Johan van der Vlag, Joshua M. Thurman, Dorin-Bogdan Borza

**Affiliations:** ^1^Division of Nephrology, Department of Medicine, Vanderbilt Medical Center, Nashville, TN, United States; ^2^Vanderbilt Center for Kidney Disease, Vanderbilt Division of Nephrology, Nashville, TN, United States; ^3^Renal Division, Washington University School of Medicine, St. Louis, MO, United States; ^4^Division of Nephrology, Boston University Medical Center, Boston, MA, United States; ^5^Department of Nephrology, Radboud University Medical Center, Nijmegen, Netherlands; ^6^Department of Medicine, University of Colorado School of Medicine, Aurora, CO, United States; ^7^Department of Microbiology, Immunology and Physiology, Meharry Medical College, Nashville, TN, United States

**Keywords:** membranous nephropathy, glomerulonephritis, albuminuria, alternative pathway, membrane attack complex, factor B, complement C5, mouse models

## Abstract

Membranous nephropathy is an immune kidney disease caused by IgG antibodies that form glomerular subepithelial immune complexes. Proteinuria is mediated by complement activation, as a result of podocyte injury by C5b-9, but the role of specific complement pathways is not known. Autoantibodies-mediating primary membranous nephropathy are predominantly of IgG4 subclass, which cannot activate the classical pathway. Histologic evidence from kidney biopsies suggests that the lectin and the alternative pathways may be activated in membranous nephropathy, but the pathogenic relevance of these pathways remains unclear. In this study, we evaluated the role of the alternative pathway in a mouse model of membranous nephropathy. After inducing the formation of subepithelial immune complexes, we found similar glomerular IgG deposition in wild-type mice and in factor B-null mice, which lack a functional alternative pathway. Unlike wild-type mice, mice lacking factor B did not develop albuminuria nor exhibit glomerular deposition of C3c and C5b-9. Albuminuria was also reduced but not completely abolished in C5-deficient mice. Our results provide the first direct evidence that the alternative pathway is necessary for pathogenic complement activation by glomerular subepithelial immune complexes and is, therefore, a key mediator of proteinuria in experimental membranous nephropathy. This knowledge is important for the rational design of new therapies for membranous nephropathy.

## Introduction

Membranous nephropathy (MN), one of the leading causes of nephrotic syndrome in adults, is an antibody-mediated kidney disease clinically characterized by proteinuria, often heavy and persistent. MN is a disease of heterogeneous etiology, defined histologically by immune complexes deposited on the subepithelial side of glomerular basement membrane (GBM), together with GBM thickening and podocyte foot process effacement, but little glomerular inflammation. The most prevalent form is primary MN, now understood as an autoimmune disease in which IgG autoantibodies (predominantly of IgG4 subclass) form subepithelial immune complexes with autoantigens expressed on podocyte cell surface (1, 2). About 70% of patients with primary MN have autoantibodies targeting phospholipase A2 receptor (PLA2R), and an additional 3–5% have autoantibodies targeting thrombospondin type-1 domain-containing 7A (THSD7A) (3, 4). Secondary MN can occur when circulating antibodies bind to antigens planted in the subepithelial space, such as cationic bovine serum albumin of dietary origin (5).

In the current paradigm for the pathogenesis of MN, complement activation is a major effector mechanism of subepithelial immune complexes (1, 6). Complement activation is initiated by three canonical pathways. The classical pathway is triggered by immune complexes and the lectin pathway—by certain danger patterns, while the alternative pathway (AP) is constitutively active and self-amplifies on foreign surfaces. The AP also amplifies activation that is initiated through the other two pathways. All three pathways converge toward the assembly of C3 and C5 convertases, which generate pro-inflammatory anaphylatoxins (C3a, C5a), opsonins that mediate immune adhesion (C3b, iC3b), and the membrane attack complex (C5b-9), which lyses cells. In human MN, C3 fragments and C5b-9 are present alongside IgG in subepithelial deposits, while urinary excretion of sC5b-9 associates with immune disease activity (7–9). Studies in passive Heymann nephritis, a faithful rat model of MN, have specifically implicated C5b-9 as a key mediator of podocyte injury and proteinuria (10, 11). However, the role of different complement pathways upstream of C5 activation in human and experimental MN remains largely unknown.

How immune complexes activate complement in MN remains a conundrum because the autoantibodies implicated in primary MN are predominantly of IgG4 subclass (6, 12–14). Although immune complexes typically bind C1q and activate the classical pathway, IgG4 does not bind C1q and is considered unable to activate complement—at least not *via* the classical pathway (15–17). In kidney biopsies from patients with primary MN, glomerular staining for C1q is almost always weak or absent, while staining for mannan-binding lectin (MBL) and C4d is usually positive (except in patients with MBL deficiency), consistent with the activation of the lectin pathway (18–20). Beck and Salant proposed that the lectin pathway may be activated by IgG4 glycoforms that lack terminal galactose and sialic acid, and, therefore, can bind MBL (1, 6). However, the occurrence of primary MN in patients with MBL deficiency shows that the lectin pathway is not absolutely required (21). Glomerular deposition of properdin and factor B—which is indicative of the AP activation—is also common in MN (18, 21), but the pathogenic relevance of this pathway is not known.

The goal of this study was to determine the contribution of the AP to glomerular injury and proteinuria mediated by subepithelial immune complexes. For this purpose, we used a mouse model that recapitulates clinical and morphologic features of human MN (22–25) and which was found to exhibit proteinuria exacerbated by C5 activation. Using Cfb^−/−^ mice, which lack factor B (an essential component of the AP), we found that the absence of a functional AP prevented complement activation by subepithelial immune complexes (as assessed from the glomerular deposition of C3c and C5b-9) and abolished proteinuria. These findings provide the first direct evidence implicating the AP activation in the pathogenesis of MN. This knowledge may provide a framework for developing new therapeutic strategies for MN.

## Materials and Methods

### Materials

The recombinant non-collagenous (NC1) domain of human α3(IV) collagen (rh-α3NC1) was expressed in HEK293 cells and purified as described (26).

### Animal Experiments

DBA/1J (D1), DBA/2J (D2), and C57Bl/6J (B6) mice were purchased from The Jackson Laboratory (Bar Harbor, ME, USA). Breeding pairs of Cfb^−/−^ mice backcrossed on the B6 background for more than nine generations (B6.Cfb^−/−^) were obtained from Dr. Joshua Thurman and maintained by homozygous breeding. D1.Cfb^−/−^ mice were generated by backcrossing onto the D1 background for four generations and then intercrossing F4 heterozygous mice. Mice were housed in a specific pathogen-free facility with free access to food and water. The study was carried out in accordance with the recommendations of the National Institutes of Health Guide for Care and Use of Laboratory Animals and the protocol was approved by the local Institutional Animal Care and Use Committee.

To induce experimental membranous nephropathy, mice (6–10 weeks old, both male and female) were immunized subcutaneously at two sites on the back with rh-α3NC1 antigen (30 µg in 50 µL sterile phosphate-buffered saline) emulsified in an equal volume of Complete Freund’s Adjuvant (Sigma, St. Louis, MO, USA). D1 and D2 mice received one booster immunization with the rh-α3NC1 antigen in Incomplete Freund’s Adjuvant (Sigma, St. Louis, MO, USA) on day 21 after the first immunization. B6 mice were boosted four times with rh-α3NC1 in Incomplete Freund’s Adjuvant, at 10 days interval starting on day 14 after the first immunization. As negative controls, some mice were immunized with adjuvant alone in each experiment. Unless otherwise indicated, mice were euthanized at 8 weeks (for D1 and D2 mice) or 12 weeks (for B6 mice) after the initial immunization, and tissues and blood were collected for further evaluations.

### Evaluation of Kidney Function

Spontaneously voided spot urine samples were collected by placing mice over 96-well microtiter plates and then analyzed as follows. Urinary albumin was measured by capture ELISA using a mouse albumin quantitation kit (Bethyl, Montgomery, TX, USA). Urine creatinine and blood urea nitrogen levels were measured using Infinity creatinine and urea liquid stable reagents (Thermo Fisher Scientific, Middletown, VA, USA), according to the manufacturer’s protocols. To account for urine tonicity, albuminuria was expressed as urinary albumin-to-creatinine ratio (ACR).

### Evaluation of Mouse IgG Autoantibody Production

Sera were assayed for the presence of IgG antibodies to rh-α3NC1 by ELISA. Briefly, 96-well microtiter plates (Nunc MaxiSorp) were coated overnight with rh-α3NC1 (100 ng per well) in carbonate-bicarbonate buffer, pH 9.6. After blocking with 1% bovine serum albumin, the wells were incubated for 1 h with mouse sera diluted 1/5,000 for detection of total IgG, 1/2,000 for detection of IgG1, or 1/500 for detection of IgG2a and IgG2b. Secondary antibodies were alkaline phosphatase-conjugated goat anti-mouse IgG (Rockland Immunochemicals, Gilbertsville, PA, USA) and horseradish peroxidase-conjugated goat anti-mouse IgG1, IgG2a, IgG2b, or IgG2c (Bethyl, Montgomery, TX, USA). Plates were developed with p-nitrophenol phosphate or tetramethylbenzidine (Sigma, St. Louis, MO, USA) as substrate, and absorbance was read at 405 nm with a SpectraMax ELISA plate reader (Molecular Devices, Sunnyvale, CA, USA).

### Renal Histopathology and Immunofluorescence Microscopy

For light microscopy, portions of mouse kidneys were fixed in 10% buffered formalin, dehydrated through a graded ethanol series, and embedded in paraffin. Paraffin sections (2 µm thick) were stained with periodic-acid Schiff reagent. Transmission electron microscopy was performed as described (27). For immunofluorescence, portions of mouse kidneys embedded in OCT were snap-frozen in isopentane and stored at −70°C. Cryosections cut at a thickness of 5 µm were fixed in cold acetone for 10 min. Mouse IgG was visualized using FITC-conjugated goat anti-mouse IgG (BD Bioscience Pharmingen, San Jose, CA, USA). Complement C3 was visualized using FITC-conjugated goat anti-mouse C3c (Nordic Immunology, Tilburg, Netherlands). Kidney deposition of rh-α3NC1 was visualized with rat IgG mAb RH34 (a gift from Dr. Yoshikazu Sado, Shigei Medical Research Institute, Japan), which is specific for human but not mouse α3NC1 (28). Mouse C5b-9, properdin, factor H, and C4 were visualized using rabbit anti-C5b9 (Abcam; Cambridge, MA, USA), rabbit anti-properdin (Santa Cruz, CA, USA), rat IgG anti-mouse C4d mAb (HyCult, Netherlands), and rat IgG anti-mouse factor H mAb (MAB4999, R&D Systems, Minneapolis, MN, USA). Nephrin was visualized using guinea pig anti-nephrin (Progen, Germany). Heparan sulfate chains were visualized with mouse IgM mAb JM403 (29). Agrin was visualized using a rabbit anti-agrin polyclonal antibody (kindly provided by Dr. Takako Sasaki, Oita University, Japan), as previously described (27). Secondary antibodies were Alexa Fluor 488-conjugated goat anti-rabbit IgG, goat anti-rat IgG, donkey anti-guinea pig IgG, goat anti-mouse IgM (Invitrogen, Carlsbad, CA, USA), and FITC-goat anti-rat IgG (BD Bioscience Pharmingen, San Jose, CA, USA). Sections were mounted with anti-fade reagent (Invitrogen, Carlsbad, CA, USA), then coverslipped and observed using a fluorescence microscope (Nikon Eclipse E800). Photomicrographs were captured with a digital camera attached to the microscope, using the same exposure settings for each primary antibody. For quantitative analyses, images were analyzed with Image J software, as described (30). All sections from one experiment were stained and analyzed at the same time.

### *In Vitro* Complement Activation

Fresh frozen normal mouse serum, collected from DBA/1 mice, and stored in aliquots at −70°C, was used as a source of complement. Cryosections of mouse kidneys fixed in cold acetone were incubated overnight at 37°C with normal mouse serum diluted 1:3 in veronal buffered saline (Sigma, St. Louis, MO, USA) containing 0.1% Tween 20 and supplemented with: (a) 2.5 mM calcium chloride and 0.7 mM magnesium chloride; or (b) 2.5 mM magnesium chloride and 6.2 mM EGTA; or (c) 25 mM EDTA. Complement activation was visualized by staining with FITC-conjugated goat anti-mouse C3c, as described above.

### Statistical Analyses

Analyses were performed using GraphPad Prism 7.00 software (San Diego, CA, USA). ACRs were log transformed. The significance of differences was determined by unpaired *t* test or by one-way analysis of variance (ANOVA) with Dunnett’s correction for multiple comparisons. A value of *P* < 0.05 was considered statistically significant. Values are presented as means ± SEM.

## Results

### Complement C5 Deficiency Ameliorates Albuminuria Induced by Subepithelial Immune Complexes in Mice Immunized With α3NC1

DBA/1 (D1) mice immunized with α3NC1 develop kidney disease recapitulating clinical and morphologic features of human MN (22–25). This model exhibits proteinuria associated with subepithelial immune complexes and glomerular deposition of IgG, C3, and C5b-9, but the role of complement activation in proteinuria is not known. We reasoned that if C5b-9 is pathogenic in this mouse model (analogous to the rat Heymann nephritis model), then, proteinuria would be ameliorated by the genetic deficiency of complement C5, which occurs naturally in several inbred strains of mice (31). To test this conjecture, we induced experimental MN in C5-deficient DBA/2 (D2) mice, which are nearly 95% genetically identical to C5-sufficient D1 mice (32).

**Figure 1:**
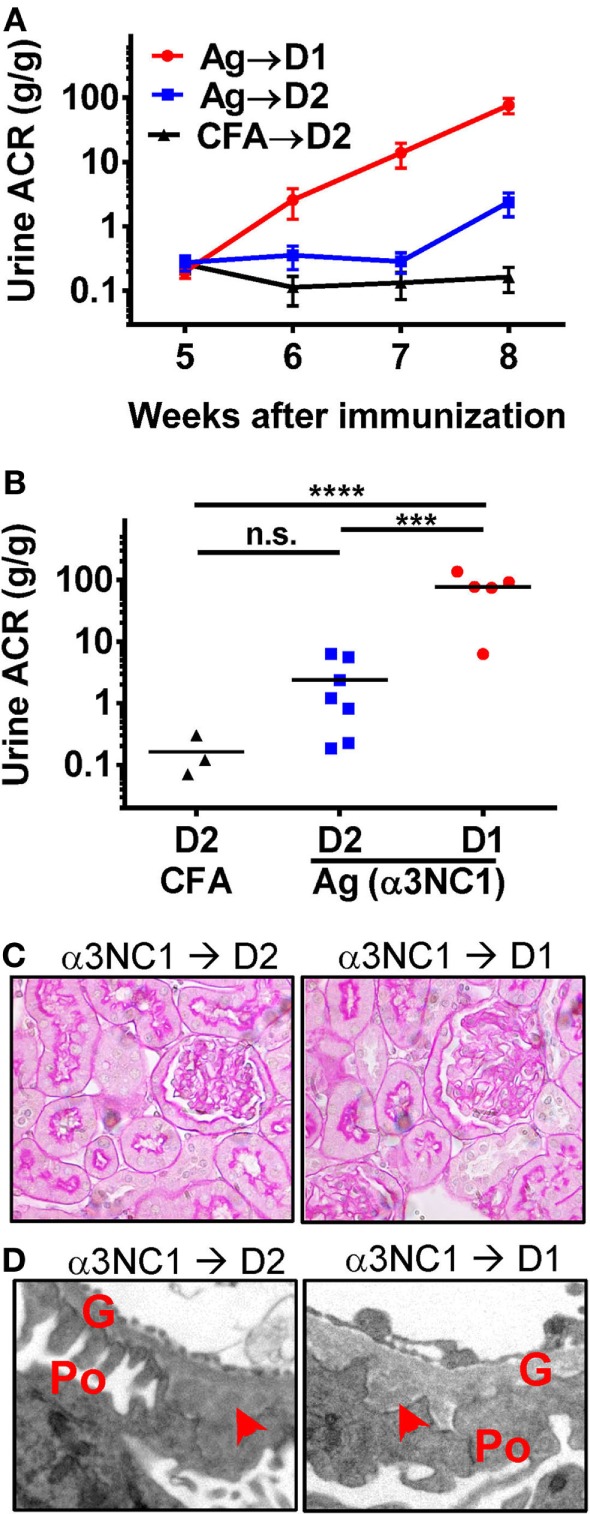
C5 deficiency protects against albuminuria in experimental MN. **(A)** Time course of urinary albumin-to-creatinine ratio (ACR) in D1 mice (circles) and D2 mice (squares) immunized with α3NC1 (*N* = 5–7 per group). Control D2 mice (triangles) were immunized with adjuvant alone (*N* = 3). Shown are means and SEM. **(B)** Scatterplot depicting the final ACR values (at week 8). The significance of differences among groups was analyzed by one-way ANOVA with Dunnett’s correction for multiple comparisons. ***P < 0.001, ****P < 0.0001. n.s., not significant. **(C)** Light microscopy shows normal appearance of glomeruli and tubules in α3NC1-immunized D2 and D1 mice (periodic acid–Schiff staining; original magnification 400×). **(D)** Transmission electron microscopy shows subepithelial electron dense deposits (arrowhead), areas of glomerular basement membrane (G) thickening, and effacement of podocyte (Po) foot processes. Original magnification 7,500×.

Compared to D1 mice, D2 mice immunized with α3NC1 developed much milder albuminuria (Figure 1A). At week 8 after the first immunization, the endpoint for this experiment, the urine ACR in α3NC1-immunized D2 mice (2.4 ± 0.96) was significantly lower (*P* < 0.001) than that in α3NC1-immunized D1 mice (77.1 ± 20.8), albeit greater than in control D2 mice immunized with adjuvant alone (ACR 0.16 ± 0.07) (Figure 1B). Blood urea nitrogen levels did not increase over the baseline (mean values at week 0 and at week 8 were 23.1 and 20.3 mg/dL for D1 mice, 27.8 and 25.4 mg/dL for D2 mice), indicating that the renal function did not decline during the duration of the experiment. Serum levels of mouse IgG anti-α3NC1 antibodies were similar in D1 and D2 mice (data not shown). Kidneys were collected at 8 weeks post-immunization for morphologic evaluation. By light microscopy, kidneys from α3NC1-immunized mice appeared relatively normal, with little glomerular inflammation (Figure 1C); however, electron microscopy revealed areas of thickened GBM surrounding subepithelial deposits and podocyte foot process effacement (Figure 1D).

**Figure 2:**
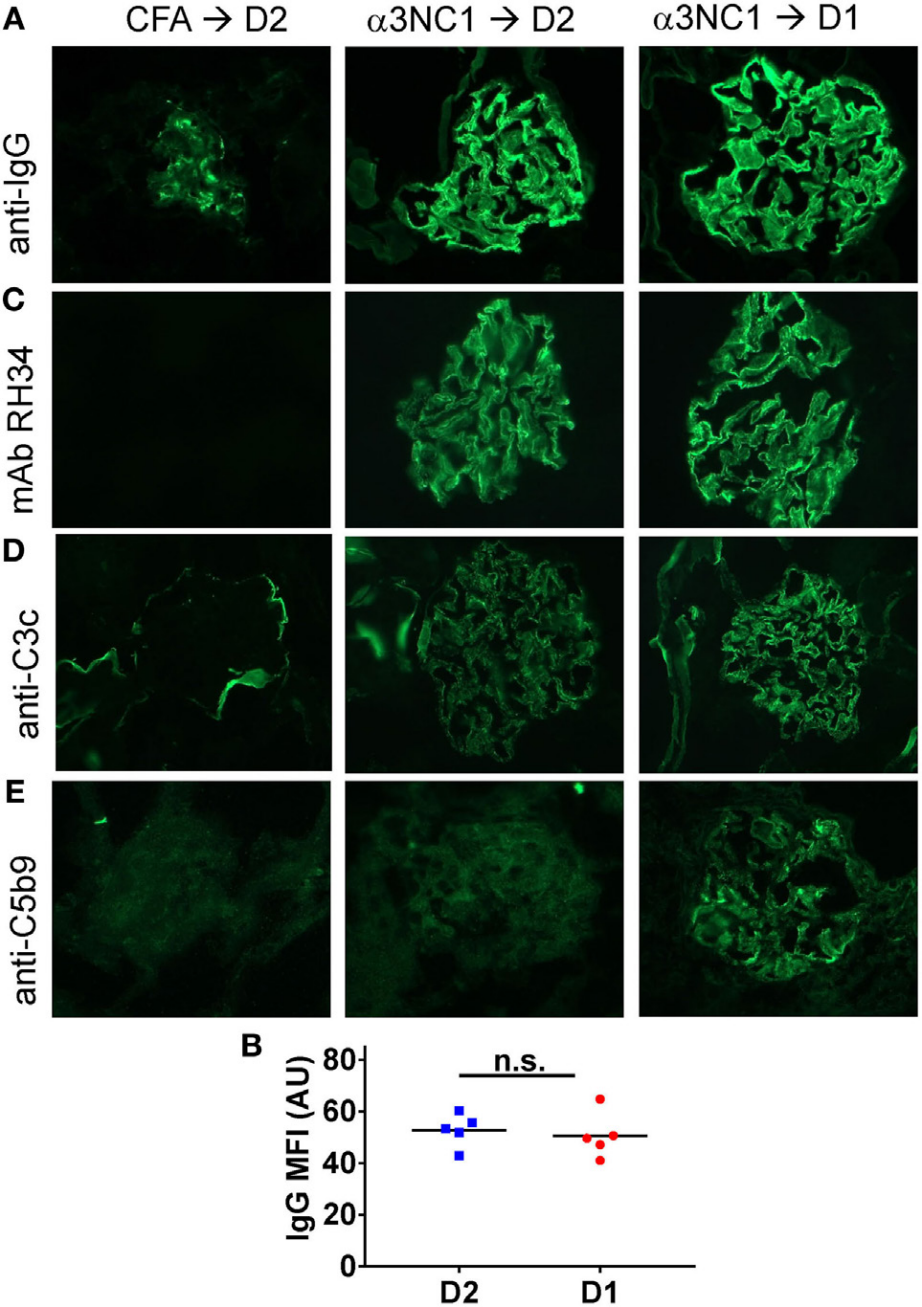
Analysis of glomerular immune complexes and complement deposition in D1 and D2 mice immunized with α3NC1. Kidneys were collected at week 8 post-immunization from adjuvant immunized D2 mice (left), α3NC1-immunized D2 mice (middle), and α3NC1-immunized D1 mice (right). **(A)** Direct immunofluorescence staining shows mouse IgG binding along the glomerular basement membrane (GBM) in α3NC1-immunized D1 and D2 mice. Control mice show non-specific mesangial deposition of mouse IgG. **(B)** Mean fluorescence intensity (MFI) of IgG staining, expressed in arbitrary units (AU), was compared in α3NC1-immunized D2 and D1 mice. The difference was not significant (n.s.) by *t* test. **(C)** Indirect immunofluorescence staining with mAb RH34 shows GBM deposition of exogenous antigen in all α3NC1-immunized mice, which is absent in adjuvant-immunized mice. **(D)** Direct immunofluorescence staining reveals C3c deposition along the capillary loops in α3NC1-immunized D1 and D2 mice. In control mice, C3c staining is positive in the Bowman’s capsule and kidney tubules. **(E)** Indirect immunofluorescence shows capillary loop staining for C5b-9 in α3NC1-immunized D1 mice. A weak background of non-specific immunofluorescence is observed in adjuvant-immunized and α3NC1-immunized D2 mice. Original magnification 400×.

Immunofluorescence microscopy showed mouse IgG staining along the GBM in all α3NC1-immunized mice (Figure 2A). The IgG staining intensity was similar in D1 and D2 mice (Figure 2B). Staining with an antibody that binds to human but not mouse α3NC1 (mAb RH34) showed capillary loop deposition of exogenous antigen in α3NC1-immunized but not control mice (Figure 2C). GBM staining for C3c, which indicates recent complement activation, was also found in all α3NC1-immunized mice (Figure 2D). However, glomerular deposition of C5b-9 was only found in α3NC1-immunized D1 mice (Figure 2E), as expected based on the C5 deficiency in D2 mice. A decrease in nephrin staining in α3NC1-immunized D1 mice compared to D2 mice confirmed an injured podocyte phenotype (not shown). These results imply that C5 activation exacerbates proteinuria induced by subepithelial immune complexes in α3NC1-immunized mice, presumably as a result of podocyte injury by C5b-9.

### Evidence of the AP Activation by Subepithelial Immune Complexes

**Figure 3:**
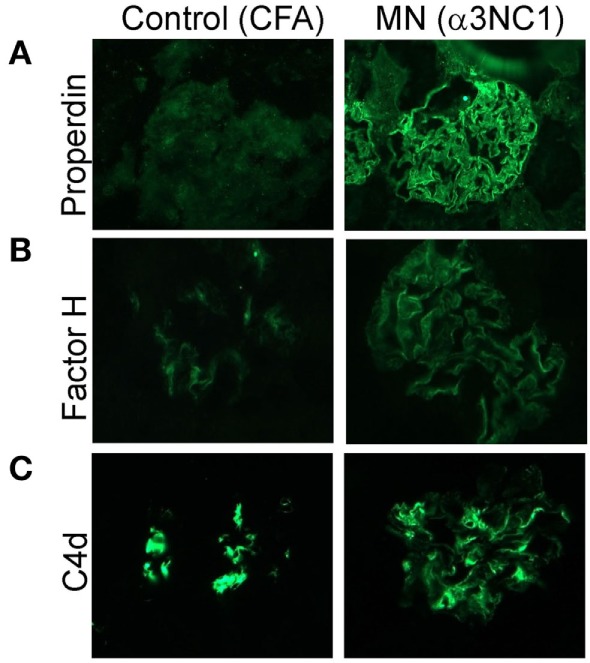
Histologic evidence of AP activation in experimental MN. Glomerular deposition of properdin **(A)**, factor H **(B)**, and C4d **(C)** was evaluated by indirect immunofluorescence staining of frozen kidney sections from α3NC1-immunized D1 mice and adjuvant-immunized control mice. Properdin and factor H staining along the capillary loops was found in α3NC1-immunized D1 mice, but not in adjuvant-immunized mice. Mesangial C4d staining was found in all mice, while weaker segmental staining for C4d along capillary loops was also present in α3NC1-immunized mice.

To determine which complement pathways may be activated by subepithelial immune complexes, we assessed glomerular deposition of properdin, factor H, and C4d. Properdin and factor H are positive and negative regulators of the AP, respectively, while C4d is a marker of the classical and lectin pathway activation. Capillary loop staining for both properdin (Figure 3A) and factor H (Figure 3B) was found in α3NC1-immunized D1 mice but not in control mice. All mice had staining for C4d in a non-specific mesangial pattern (Figure 3C), also commonly seen in normal human glomeruli (33). In addition, α3NC1-immunized mice also had segmental staining for C4d along the capillary loops, suggesting that subepithelial immune complexes formed in these mice activate multiple complement pathways, similar to what is observed in human MN (18). While these results are indicative of the AP activation, the pathogenic relevance of the AP cannot be inferred from morphologic findings alone.

### B6.Cfb^−/−^ Mice Immunized With α3NC1 Develop Subepithelial Immune Complexes but Are Protected Against Albuminuria and Do Not Exhibit Glomerular Complement Deposition

**Figure 4:**
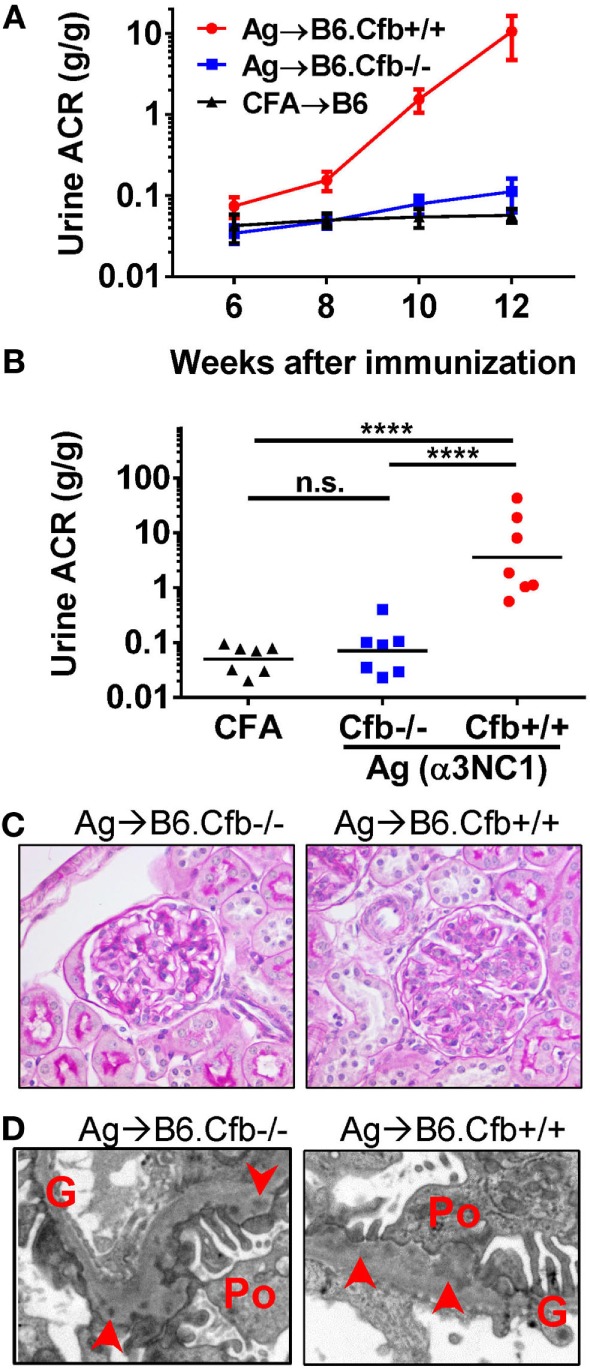
B6.Cfb^−/−^ mice immunized with α3NC1 are protected against albuminuria despite developing subepithelial immune complexes. **(A)** Time course of urinary albumin-to-creatinine ratio (ACR) in B6.Cfb^+/+^ mice (circles) and B6.Cfb^−/−^ mice (squares) immunized with α3NC1 (*N* = 7 per group). Control B6 mice (triangles), including both genotypes, were immunized with adjuvant alone (*N* = 7). Shown are means and SEM. **(B)** Scatterplot depicts the ACR values at the endpoint of this experiment (week 12). The significance of differences among groups was analyzed by one-way ANOVA with Dunnett’s correction for multiple comparisons. ***P < 0.001, ****P < 0.0001. n.s., not significant. **(C)** Morphology of kidneys from α3NC1-immunized B6.Cfb^−/−^ and B6.Cfb^+/+^ mice appears normal by light microscopy (periodic acid–Schiff staining, original magnification 400×). **(D)** Transmission electron microscopy shows subepithelial electron dense deposits (arrowhead), expansion of the glomerular basement membrane (G), and podocyte (Po) foot process effacement. Original magnification 7,500×.

The absence of a functional AP in Cfb^−/−^ mice afforded a strategy to investigate the role of the AP in experimental MN. We initially obtained Cfb^−/−^ mice backcrossed on the B6 background for nine generations (B6.Cfb^−/−^), which allowed us to compare the course of albuminuria induced by α3NC1 immunization in B6.Cfb^−/−^ mice to congenic wild-type B6 mice (B6.Cfb^+/+^) (Figure 4A). At the endpoint in this experiment (i.e., at 12 weeks after the initial immunization), the urinary ACR in α3NC1-immunized B6.Cfb^−/−^ mice (0.11 ± 0.05) was similar to that in adjuvant-immunized B6 mice (0.057 ± 0.011); both were significantly lower (*P* < 0.0001) than the urine ACR in α3NC1-immunized B6.Cfb^+/+^ mice (10.6 ± 5.9) (Figure 4B). Two mice from each group were observed for an additional 4 weeks; albuminuria further increased in Cfb^+/+^ mice but not in Cfb^−/−^ mice. In both groups of α3NC1-immunized B6 mice, kidney morphology mice appeared relatively normal by light microscopy (Figure 4C). Electron microscopy showed features of MN including subepithelial deposits, GBM expansion, and foot process effacement (Figure 4D).

**Figure 5:**
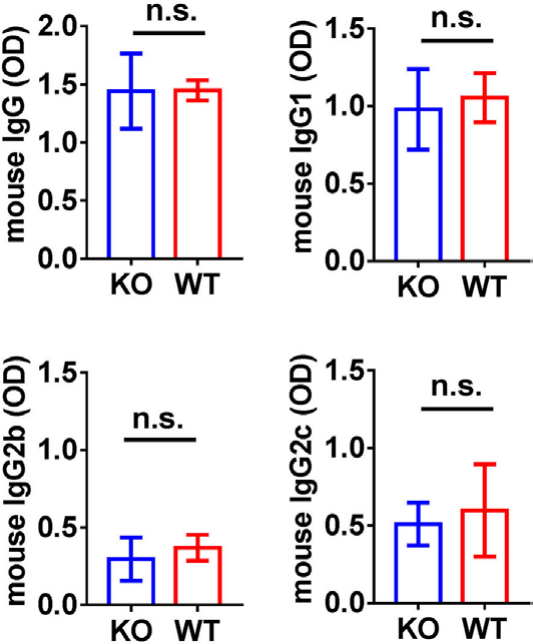
Analysis of circulating anti-α3NC1 mouse IgG antibodies. The levels of circulating mouse IgG, IgG1, IgG2b, and IgG2c antibodies binding to rh-α3NC1 were measured by indirect ELISA. Mouse sera were diluted 1/5,000 for total IgG, 1/2,000 for IgG1, and 1/500 for IgG2b and IgG2c. The significance of differences between Cfb^−/−^ (KO) and Cfb^+/+^ (WT) mice was evaluated by *t*-test (n.s., not significant).

**Figure 6:**
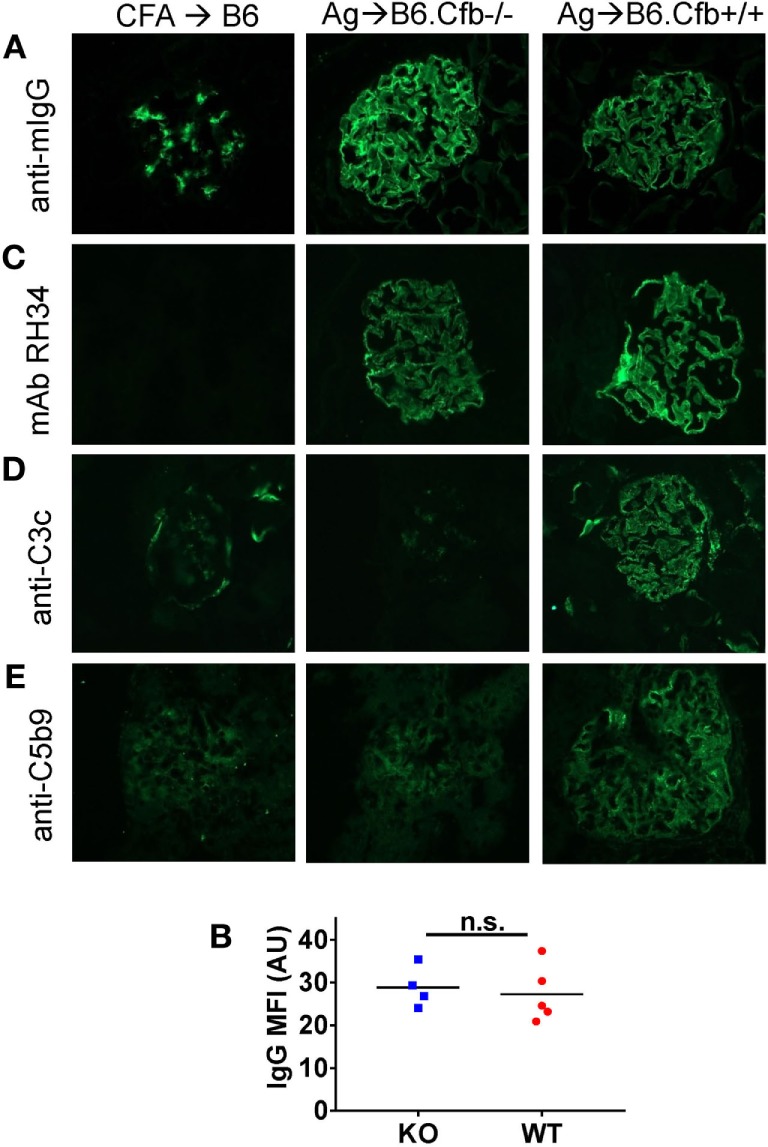
Analysis of glomerular immune complexes and complement deposition in B6 mice immunized with α3NC1. Kidneys from adjuvant-immunized B6 mice (left), α3NC1-immunized B6.Cfb^−/−^ mice (middle), and α3NC1-immunized B6.Cfb^−/−^ mice (right) were collected at week 12 after the initial immunization. **(A)** Immunofluorescence staining for mouse IgG shows glomerular basement membrane (GBM) staining in α3NC1-immunized B6.Cfb^−/−^ and B6.Cfb^+/+^ mice. Adjuvant-immunized mice show non-specific mesangial IgG deposition. **(B)** Mean fluorescence intensity (MFI) of IgG staining, expressed in arbitrary units (AU), was compared in α3NC1-immunized B6.Cfb^−/−^ and B6.Cfb^+/+^ mice. Significance was evaluated by *t* test (n.s., not significant). **(C)** Staining with mAb RH34 shows GBM deposition of exogenous antigen in α3NC1 immunized B6.Cfb^−/−^ and B6.Cfb^+/+^ mice, which is absent in control mice. **(D)** Direct immunofluorescence staining reveals C3c deposition in a capillary loop pattern in α3NC1-immunized B6.Cfb^+/+^ mice. C3c staining is absent in B6.Cfb^−/−^ mice. **(E)** Indirect immunofluorescence shows C5b-9 deposition along the GBM in α3NC1-immunized B6.Cfb^+/+^ mice, but not B6.Cfb^−/−^ mice. Original magnification 400×.

Humoral immune responses to α3NC1 were comparable in immunized B6.Cfb^−/−^ and B6.Cfb^+/+^ mice, as shown by similar levels of circulating mouse IgG, IgG1, IgG2b, and IgG2c anti-α3NC1 antibodies (Figure 5). Immune complexes and complement deposition were evaluated by immunofluorescence microscopy (Figure 6). GBM staining for mouse IgG was found in all α3NC1-immunized mice (Figure 6A), with similar intensity in B6.Cfb^−/−^ and B6.Cfb^+/+^ mice (Figure 6B). GBM deposition of exogenous antigen in α3NC1-immunized mice was indicated by positive staining with mAb RH34 (Figure 6C). Deposition of C3c along the GBM was found in α3NC1-immunized Cfb^+/+^ mice but was completely absent in immunized Cfb^−/−^ mice (Figure 6D); as also found for C5b-9 (Figure 6E). These results indicate that the absence of factor B does not affect the formation of subepithelial immune complexes but prevents glomerular complement activation and abolishes proteinuria induced by immunization with α3NC1.

### Absence of Proteinuria and Glomerular Complement Deposition in D1.Cfb^−/−^ Mice Immunized With α3NC1 Corroborates the Pathogenic Role of the AP in Experimental MN

We sought to verify whether the ablation of the AP also prevents albuminuria in D1 mice, which are more susceptible to MN. Results in B6 mice cannot be directly compared to those in D1 and D2 mice because B6 mice are more resistant to experimental kidney disease and develop milder proteinuria requiring repeated immunizations with α3NC1. After backcrossing to D1 for four generations, we generated D1.Cfb^−/−^ mice, which had about 94% D1 genetic background (comparable to almost 95% genetic similarity between D1 and D2 mice).

**Figure 7:**
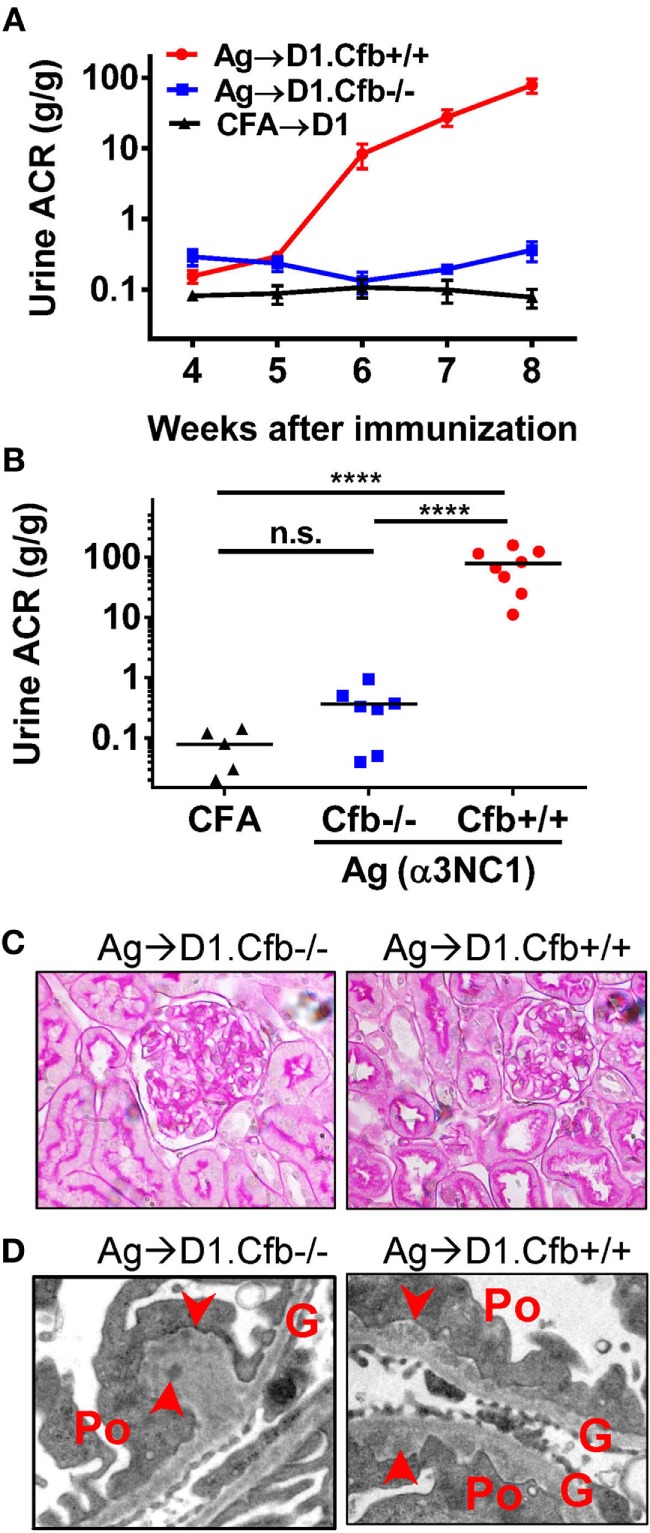
D1.Cfb^−/−^ mice immunized with α3NC1 are protected against albuminuria despite developing subepithelial immune complexes. **(A)** Time course of urinary albumin-to-creatinine ratio (ACR) in D1.Cfb^+/+^ mice (circles) and D1.Cfb^−/−^ mice (squares) immunized with α3NC1 (*N* = 7–8 per group). Control D1 mice (triangles), including both genotypes, were immunized with adjuvant alone (*N* = 5). Shown are means and SEM. **(B)** Scatterplot depicts the ACR values at the last time point (week 8). The significance of differences among groups was analyzed by one-way ANOVA with Dunnett’s correction multiple comparisons. ***P < 0.001, ****P < 0.0001, n.s., not significant. **(C)** Morphology of kidneys from α3NC1-immunized D1.Cfb^−/−^ (left) and D1.Cfb^+/+^ mice (right) appeared normal by light microscopy (periodic acid–Schiff staining, original magnification 400×). **(D)** Transmission electron microscopy shows subepithelial electron dense deposits (arrowhead), thickening of the glomerular basement membrane (G), and podocyte (Po) foot process effacement. Original magnification 15,000×.

**Figure 8:**
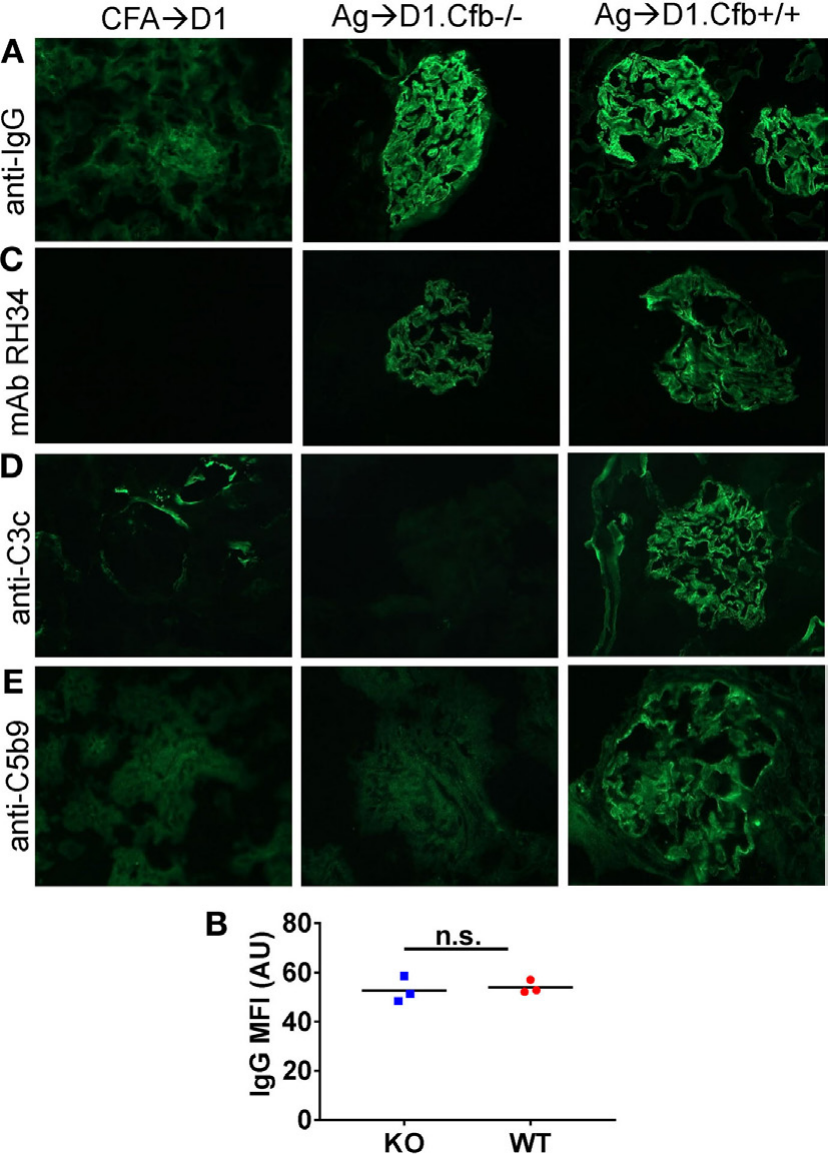
Analysis of glomerular immune complexes and complement deposition in D1 mice immunized with α3NC1. Kidneys were collected from adjuvant immunized D1 mice (left), α3NC1-immunized D1.Cfb^−/−^ mice (middle), and α3NC1-immunized D1.Cfb^−/−^ mice (right) at week 8 after the initial immunization. **(A)** Immunofluorescence staining for mouse IgG shows glomerular basement membrane (GBM) staining in α3NC1-immunized D1.Cfb^−/−^ and D1.Cfb^+/+^ mice. Adjuvant-immunized control mice show non-specific mesangial IgG deposition. **(B)** Mean fluorescence intensity (MFI) of IgG staining, expressed in arbitrary units (AU), was compared in α3NC1-immunized D1.Cfb^−/−^ and D2.Cfb^+/+^ mice. The difference was not significant (n.s.) by *t* test. **(C)** Staining with mAb RH34 shows the GBM deposition of exogenous antigen in both groups of α3NC1-immunized mice, which is absent in control mice. **(D)** Direct immunofluorescence staining reveals capillary loop deposition of C3c in α3NC1-immunized D1.Cfb^+/+^ mice, while C3c staining is absent in D1.Cfb^−/−^ mice. **(E)** Indirect immunofluorescence shows C5b-9 deposition along the GBM in α3NC1-immunized D1.Cfb^+/+^ mice but not D1.Cfb^−/−^ mice. Original magnification 400×.

D1.Cfb^−/−^ mice were resistant to development of albuminuria induced by α3NC1 immunization (Figure 7A). At the final time point (at week 8 post-immunization), urine ACR in α3NC1-immunized D1.Cfb^−/−^ mice (0.36 ± 0.12) was similar to that in adjuvant-immunized D1 mice (0.078 ± 0.023); both were significantly lower (*P* < 0.0001) than urine ACR in α3NC1-immunized D1.Cfb^+/+^ mice (78.8 ± 18.1) (Figure 7B). Morphologic analyses of the kidneys collected at week 8 largely recapitulated the findings from B6 mice. Light microscopy did not show major glomerular abnormalities (Figure 7C), while electron microscopy showed subepithelial deposits with GBM thickening and effaced podocyte foot processes (Figure 7D). GBM staining for mouse IgG was found in all α3NC1-immunized but not control mice (Figure 8A). The IgG staining intensity was similar in D1.Cfb^−/−^ and D1.Cfb^+/+^ mice (Figure 8B). Staining with mAb RH34 showed GBM deposition of exogenous antigen in these mice (Figure 8C). Capillary loop deposition of C3c (Figure 8D) and C5b-9 staining (Figure 8E) was observed in α3NC1-immunized D1.Cfb^+/+^ mice but was absent from α3NC1 immunized D1.Cfb^−/−^ mice. These results confirm that the AP is essential for pathogenic complement activation by subepithelial immune complexes and further show that the ablation of the AP abolishes proteinuria to a greater extent than C5 deficiency under comparable experimental conditions.

### Subepithelial Immune Complexes Activate Complement *In Vitro via* the AP

**Figure 9:**
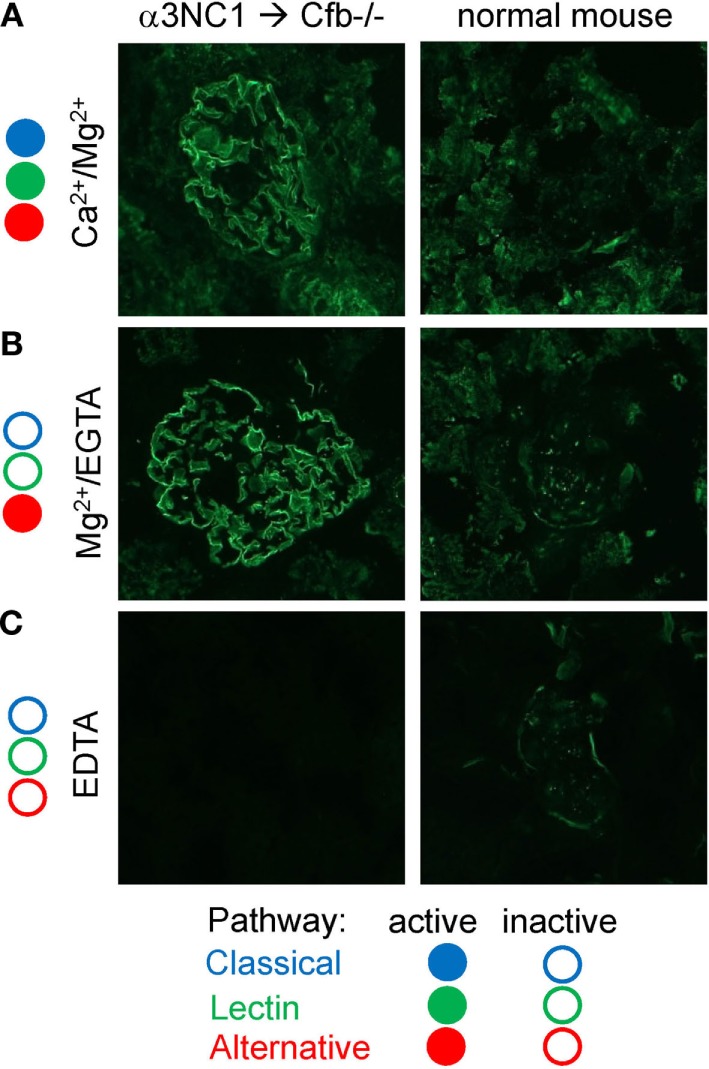
*In vitro* complement activation by glomerular immune complexes from α3NC1-immunized Cfb^−/−^ mice. Immunofluorescence analysis of C3c deposition onto kidneys cryosections from α3NC1-immunized D1.Cfb^−/−^ mice (left) and control non-immunized wild-type DBA/1 mice (right), after incubation with 33% normal mouse serum in buffer containing Ca^2+^ and Mg^2+^ **(A)**, Mg^2+^ and EGTA **(B)**, or EDTA **(C)**. Complement pathways active in each buffer are indicated on the left, according to the key shown at the bottom.

Since α3NC1-immunized Cfb^−/−^ mice had glomerular IgG but not C3c deposition, we investigated whether immune complexes formed in these mice can activate complement *in vitro*. To this end, kidney cryosections from D1.Cfb^−/−^ mice were incubated with normal mouse serum as a source of complement. As shown in Figure 9, glomerular capillary loops deposition of C3c was found after incubation with normal mouse serum in the presence of Ca^2+^ and Mg^2+^ (in which all three pathways are active), and also in the presence of Mg^2+^ and EGTA (conditions under which the alternative pathway is active, but the classical and lectin pathways are inhibited), but not in the presence EDTA (which inhibits all thee pathways). In control experiments using kidney cryosections from a non-immunized wild-type mouse (i.e., without glomerular IgG deposits), no C3c deposition along the capillary loops was observed under any conditions. Similar results were obtained using human serum as complement source (not shown). These results indicate that glomerular immune complexes formed *in vivo* in α3NC1-immunized Cfb^−/−^ mice have the intrinsic ability to activate complement *via* the AP.

### The Loss of GBM Heparan Sulfate in Experimental MN May Affect the Local AP Regulation

**Figure 10:**
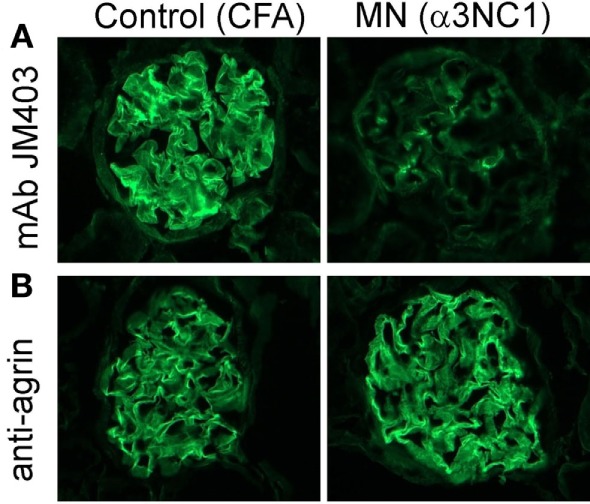
Decreased glomerular basement membrane (GBM) staining by anti-heparan sulfate mAb JM403 in experimental MN. **(A)** Immunofluorescence staining with anti-heparan sulfate mAb JM403 revealed intense staining in the GBM of control mice, which was markedly decreased in α3NC1-immunized B6.Cfb^+/+^ mice. **(B)** GBM staining for agrin core protein was similar in both groups.

Activation of the AP on extracellular matrices, which are not protected by intrinsic complement regulators, is largely controlled by factor H. Factor H selectively inhibits the AP on host surfaces by recognizing polyanions such as heparan sulfate as markers of self. The normal GBM contains heparan sulfate chains attached to the agrin core protein, but the GBM staining for heparan sulfate epitopes is markedly decreased in human MN and rat Heymann nephritis (34–36). We investigated whether similar changes occur after the induction of experimental MN in mice. Compared to adjuvant-immunized control mice, α3NC1-immunized B6 (Figure 10) and D1 mice (not shown) with proteinuria had almost complete loss of GBM staining by anti-heparan sulfate mAb JM403, while staining for agrin core protein was not changed. These results demonstrate the loss of heparan sulfate epitopes from the GBM of α3NC1-immunized mice, which may locally dysregulate the AP by reducing the recruitment of factor H (37).

## Discussion

Whereas podocyte injury by C5b-9 is a major effector mechanism of the subepithelial immune complexes that lead to proteinuria in MN, the role of specific complement pathways upstream of C5 activation remains poorly understood. Glomerular deposition of complement components specific to the AP in human MN raises the question of whether the AP activation is pathogenically relevant or just an epiphenomenon. We addressed this question by evaluating how the absence of a functional AP affects kidney disease in a mouse model of MN. We found that Cfb^−/−^ and Cfb^+/+^ mice immunized with α3NC1 developed subepithelial immune complexes, with similar IgG deposition. However, Cfb^−/−^ mice were protected against proteinuria and did not exhibit glomerular deposition of C3c and C5b-9. These results imply that the AP is required for glomerular complement activation by subepithelial immune complexes, which in turn is necessary for proteinuria. We thus provide the first direct evidence implicating the AP in the pathogenesis of experimental MN.

Complement activation can contribute to kidney disease at several levels, by causing direct injury at the glomerular filtration barrier, and also by augmenting the production of pathogenic antibodies (38). The latter appears unlikely in our model, as the levels of circulating anti-α3NC1 antibodies and kidney-bound IgG were similar in Cfb^−/−^ and wild-type mice. Hence, the more likely mechanism by which the absence of factor B protects against proteinuria is *via* the reduced complement activation at the level of C3 and C5, as implied by the absence of glomerular C3c and C5b-9 staining in Cfb^−/−^ mice despite the presence of subepithelial immune deposits. While immune complexes typically activate complement *via* the classical pathway, the AP is secondarily activated and forms an amplification loop. In a quantitative assay of complement activation initiated *via* the classical pathway (by IgM or aggregated IgG), the AP accounted for more than 80% of the C5 activation products (C5a, sC5b-9) generated in the assay (39). This amplification explains the pathogenic role of the AP in various animal models of immune complex-mediated diseases, including lupus nephritis (40–42). An increased generation of C3b would exacerbate the formation of C5b-9, which is pathogenic in MN, because C5 binding to C3b is a prerequisite for C5 activation, and very high affinity C5 convertases only form at high surface density of C3b (43, 44).

How glomerular immune complexes activate complement depends on several factors, including their ultrastructural localization and immunoglobulin composition. In human anti-GBM disease, IgG1 auto-antibodies that bind to α345(IV) collagen in the human GBM (usually specific for α3NC1) cause severe glomerulonephritis, often featuring C3 deposition along the GBM (45). By contrast, in wild-type mice, human or murine IgG antibodies that bind to α345(IV) collagen in the mouse GBM are insufficient to cause local complement activation or glomerular injury (46, 47). It is, therefore, remarkable that subepithelial immune complexes formed by mouse IgG anti-α3NC1 antibodies with exogenous rh-α3NC1 antigen (*via* a planted antigen mechanism) are able to activate complement *in vivo* and *in vitro*, even though anti-α3NC1 antibodies are predominantly of mouse IgG1 subclass. Mouse IgG1 functionally resembles human IgG4 (the predominant IgG subclass in human primary MN), in that neither binds C1q to activate the classical pathway (48). However, mouse IgG1 can activate the AP (49), as do human IgG2 and IgA (50). Whether human IgG4 can activate the AP remains an important question yet to be solved. In preliminary studies, we found that the AP amplifies *in vitro* complement activation by immune complexes formed by human anti-PLA2R IgG autoantibodies (Dorin-Bogdan Borza and Laurence H. Beck, unpublished observations).

The extent to which the AP is activated is also modulated by the local microenvironment. Although the AP is constitutively active and self-amplifies on foreign surfaces, its activation on self-surfaces is normally restricted by host complement regulatory proteins that inactivate C3 and C5 convertases. In glomeruli, podocytes are protected by membrane-bound regulators such as CR1, while plasma factor H is recruited to inhibit the AP in the GBM (51). Studies in the nephrotoxic nephritis model, in which the AP contributes to chronic but not acute kidney injury, suggest that the mechanisms controlling the AP may be impaired over time by the persistence of antibodies or by glomerular injury (52). One mechanism that could locally dysregulate the AP is the loss of heparan sulfate chains, which factor H normally recognizes as markers of self (37). Decreased GBM staining by anti-heparan sulfate mAb JM403 occurs in α3NC1-immunized mice (this study) and also in human MN (53) and a rat model of MN, active Heymann nephritis (36). The loss of GBM heparan sulfate may be the result of enzymatic cleavage by heparanase, which is often upregulated in glomerular disease (54), including in Heymann nephritis (55). It may also be due to other mechanisms (56). An apparent loss of heparan sulfate occurs in lupus nephritis due to masking by immune complexes (57). Besides factor H, heparan sulfate can also bind properdin, thus serving as a platform for AP activation on some surfaces, such as the apical surface of kidney tubules or apoptotic T cells (58, 59). In our model, given the loss of GBM heparan sulfate, glomerular deposition of properdin is more likely due to properdin binding to C3b and stabilization of the C3bBb convertase (60). Elucidating the relationships among the loss of GBM heparan sulfate, local complement regulation and proteinuria is an area for future investigations.

The role of C5b-9 in proteinuria induced by subepithelial immune complexes has been demonstrated in passive Heymann nephritis (11), but not validated in other models of MN to date. In this study, we found that C5-deficient DBA/2 mice developed significantly less proteinuria than C5-sufficient DBA/1 mice, which further supports the paradigm that C5 activation mediates glomerular injury by subepithelial immune complexes. Cleavage of C5 generates C5b, a precursor for C5b-9, and also C5a, a pro-inflammatory anaphylatoxin. Although our results cannot formally exclude a pathogenic role for C5a in the MN model used in this study, there is currently no evidence to implicate C5a as meditator of glomerular injury in MN, and inflammatory cells are usually not detected in glomeruli in MN.

An unexpected finding in this study was that the absence of factor B abolished proteinuria to a greater extent than the absence of complement C5. Under similar experimental conditions, albuminuria in C5-deficient D2 mice (ACR 2.38 ± 0.96) was reduced by a factor of about 30 compared to C5-sufficient D1 mice (ACR 77.1 ± 20.8), while albuminuria in D1.Cfb^−/−^ mice (ACR 0.36 ± 0.12) was reduced by a factor of about 200 compared to D1.Cfb^+/+^ mice (ACR 78.8 ± 18.1)—a sixfold difference. This difference may be explained by the fact the absence of C5 only prevents events downstream of C5 activation (such as C5b-9 formation), while the absence of factor B also inhibits complement activation at the level of C3. Indeed, glomerular C3c deposition was absent in Cfb^−/−^ mice but present in C5-null mice. These results suggest that complement activation at the level of C3 may also contribute to proteinuria in MN, independently of C5b-9-mediated glomerular injury.

Our study has some limitations. The role of the AP in experimental MN was only investigated using genetic approaches. Further corroboration by pharmacologic inhibition of the AP in a clinically relevant setting is desirable. This is evaluated in ongoing studies. Another potential caveat is that subepithelial immune complexes induced by immunization with α3NC1 are formed by a planted antigen mechanism, recapitulating secondary MN rather than primary MN. Nonetheless, glomerular deposition of factor B occurs not only in primary but also secondary MN (6), suggesting that the AP is involved regardless of how subepithelial immune complexes form. We expect that future studies will address the pathogenic role of the AP in emerging animal models of MN, which target autoantigens implicated in human disease. Of interest in this regard, mice injected with human anti-THSD7A antibodies develop mild proteinuria with histologic features of MN (61). However, efforts to develop models of MN targeting PLA2R have been hampered by the fact that rodents (unlike humans) do not express PLA2R on podocytes.

A better understanding of the complement-mediated mechanisms of injury in MN may help develop novel therapies for MN. MN is a common cause of nephrotic syndrome in adults, and up to 40% of patients eventually develop end-stage renal disease (62). Current therapies use non-specific immunosuppressive drugs, which have significant toxic side effects and are ineffective in about 25–30% of patients (2, 63, 64). Therapeutic inhibition of complement may be a viable approach for treating MN (65), especially in patients who do not respond to conventional therapy or have rapid deterioration of renal function. One anti-complement agent already in clinical use is eculizumab, a humanized IgG2/IgG4 anti-C5 monoclonal antibody that blocks C5 activation. An early, unpublished clinical trial of eculizumab in primary MN did not find a significant remission of proteinuria after 16 weeks of treatment, which may be explained by the insufficient dosage of the drug. Indeed, a recent study has found that C5 inhibition by eculizumab is incomplete at high C3b density (44), a setting relevant to MN. Compared to eculizumab, which does not prevent potentially harmful C3 activation, agents that inhibit complement at the level of both C3 and C5 may offer additional therapeutic benefit. The results of this study identify the AP as a novel target for therapy in MN. Inhibition of the AP has the advantage of leaving the classical and lectin pathways intact for defense against pathogens and other homeostatic functions.

In summary, our results suggest that the AP is necessary for sustained complement activation by subepithelial immune complexes, leading to glomerular deposition of C3c and C5b-9. Furthermore, the activation of the AP is essential for the development of proteinuria in experimental MN. These findings may provide a framework for the rational design of new therapies for MN.

## Ethics Statement

The study was carried out in accordance with the recommendations of the National Institutes of Health Guide for Care and Use of Laboratory Animals and the protocol was approved by the local Institutional Animal Care and Use Committee.

## Author Contributions

D-BB conceived the idea, designed the experiments, performed data analysis, and wrote the manuscript. WL and FO performed experiments and collected, assembled, and interpreted data. JM performed experiments. JV provided reagents. JT provided mice and reagents and interpreted data. LB analyzed and interpreted data. All authors contributed to editing, reviewed and approved the final manuscript.

## Conflict of Interest Statement

JT receives royalties from Alexion Pharmaceuticals, Inc. He is a consultant for AdMIRx, Inc., a company developing complement inhibitors. He also holds stock and will receive royalty income from AdMIRx. The other authors declare that the research was conducted in the absence of any commercial or financial relationships that could be construed as a potential conflict of interest.

## Abbreviations

AP, alternative pathway; GBM, glomerular basement membrane; MN, membranous nephropathy; PLA2R, phospholipase A2 receptor; THSD7A, thrombospondin type-1 domain-containing 7A.
